# Carrier trapping and confinement in Ge nanocrystals surrounded by Ge_3_N_4_

**DOI:** 10.1038/srep25449

**Published:** 2016-05-05

**Authors:** Youngsin Park, Christopher C. S. Chan, Benjamin P. L. Reid, Luke Nuttall, Robert A. Taylor, Nam-Suk Lee, Young Mi Lee

**Affiliations:** 1 School of Natural Science, Ulsan National Institute of Science and Technology (UNIST), Ulsan 44919, Korea; 2 Clarendon Laboratory, Department of Physics, University of Oxford, Oxford, OX1 3PU, UK; 3 National Institute of Nanomaterials Technology (NINT), Pohang University of Science and Technology, Pohang 37673, Korea; 4 Beamline Division, Pohang Accelerator Laboratory, Pohang 37673, Korea

## Abstract

We investigated the optical properties of Ge nanocrystals surrounded by Ge_3_N_4_. The broad emission ranging from infrared to blue is due to the dependence on the crystal size and preparation methods. Here, we report high resolution Photoluminescence (PL) attributed to emission from individual Ge nanocrystals (*nc*-Ge) spatially resolved using micro-photoluminescence and detailed using temperature and power-dependent photoluminescence studies. The measured peaks are shown to behave with excitonic characteristics and we argue that the spread of the *nc*-Ge peaks in the PL spectrum is due to different confinement energies arising from the variation in size of the nanocrystals.

Semiconductor nanostructures are arguably the most promising technology for future optoelectronics and memory device applications due to the recent rapid advances in nanoscale science and technqiues^1^,^2^,^3^. Ge is an indirect gap semiconductor, with two main electronic transitions, the first at 0.67 eV (indirect) the second at 0.8 eV (direct) and Ge nanocrystals (*nc*-Ge) are one of the candidates for such applications due to their superior charge storage performance^1^,^2^,^3^. Several methods can be used to form *nc*-Ge such as Ge ion implantation^4^, N_2_^+^ implantation^5^, SiGe oxidation^6^, thermal annealing of Ge thin films^7^,^8^, molecular beam epitaxy^9^, and co-deposition of Ge with SiO_2_^3^,^10^,^11^. Ge nanocrystals have been shown to emit photoluminescence (PL) covering a vast spectrum in both the visible, with wavelengths ranging from 460 nm to 515 nm and in the infrared at around 1600 nm^4^,^10^,^11^,^12^,^13^,^14^,^15^,^16^,^17^. PL previously reported for *nc*-Ge has been shown to give broad emission spectra in the visible range with a full width at half maximum (FWHM) of a few hundred meV. This broad spectrum indicates an ensemble effect of the nanocrystals^4^,^10^,^11^,^12^,^13^,^14^,^15^, individual *nc*-Ge emission has never been resolved. The origin of visible PL from *nc*-Ge has been discussed by Kanemitsu *et al.* who reported a change in the Ge crystalline structure at diameters below 4 nm, departing from the usual diamond structure. This new structure, beyond the 4 nm limit, is thought to allow for their observed visible luminescence with the character of a direct transition^11^. Contrary to Kanemitsu, Giri *et al.* reported observation of PL from Ge nanocrystals of a larger diameter, 4~13 nm embedded in SiO_2_^4^. In addition, S. Takeoka *et al.* measured PL that was dependent on Ge nanocrystals with average diameters of 0.9~5.3 nm embedded in SiO_2_. The PL was found to range from the near infrared at a diameter of 5.3 nm to an energy slightly larger than the band gap of bulk Ge as the nanocrystal size decreased down to 0.9 nm^17^. The broadness of the emission can be atributed to the dependence of the PL energy on nanocrystal size, but the precise explanation of the origin of this emission remains unclear. Ensemble effects have obscured the finer details of the PL spectrum, contributing to the controversy as to the origin of the Ge nanocrystal luminescence.

In this paper, Ge nanocrystals with a diameter of ~20 nm surrounded by Ge_3_N_4_ with a thickness of ~4 nm are prepared that leads to the isolation of *nc*-Ge. The formation of *nc*-Ge and Ge_3_N_4_ was confirmed by high-resolution transmission electron microscopy (HRTEM) and high-resolution X-ray photoemission (HRXPS) with synchrotron radiation. The optical properties of the *nc*-Ge were characterized by μ-PL with excitation power and temperature dependent measurements. We hypothesise that photo-carrier transfer from the trapping level of GeN to the *nc*-Ge plays an important role in the PL efficiency. Calculations have been performed to estimate the confinement energy of the *nc*-Ge.

## Results and Discussion

**Figure 1:**
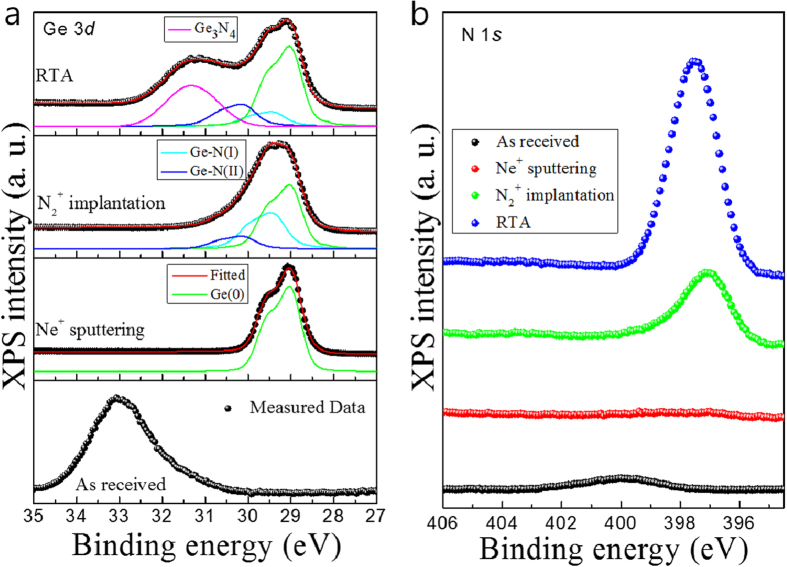
(**a**) Ge 3*d* core-levels and thier curve fittings, (**b**) N 1 *s* core-levels. The curve fitting results for the three components of the different chemical states of Ge(0), Ge-N(I), Ge-N(II), and Ge_3_N_4_ are shown.

The core-level XPS spectra of Ge 3*d* and N 1 *s* peaks are shown in Fig. 1 and corresponding valence spectra can be found in the supporting information (Fig. S2). The “as-received” Ge substrate exhibited a Ge 3*d* core-level peak corresponding to Ge oxide as observed in the bottom of Fig. 1(a). After Ne^+^ sputtering the oxide peak completely disappeared and a pure Ge 3*d* peak was observed at the peak position of the Ge 3*d*_5/2_ at 29.0 eV^18^ with 0.6 eV of spin-orbit splitting^19^. The absence of O 1 *s* and C 1 *s* core-level peaks also confirms the effective removal of these elements on the surface by sputtering. The N_2_^+^ implantation caused a significant broadening of Ge 3*d* peak to the point that the spin-orbit splitting is difficult to discern. This is due to a mixture of pure Ge and Ge nitride (such as GeN_x_) states. We also noticed that there was a shift towards higher a binding energy in the Ge 3*d* core-level spectra as the N_2_^+^ was implanted. The HRXPS data indicate bonding between N atoms and the Ge atoms. After rapid thermal annealing (RTA) treatment, a new peak was clearly observed in Ge 3*d* spectra at 31.0 eV (ΔGe_pure_ = 2.0 eV, higher chemical shift of the pure Ge 3*d* peak), indicative of the formation of Ge_3_N_4_^18^. In order to elucidate the binding energy change and chemical bonding of the Ge 3*d* core-level, deconvolution fits were performed by means of Doniach–Sunjiác functions^20^. The background due to inelastic scattering was subtracted by the Shirley (or integral) method^21^. For the Ne^+^ sputtering, a pure and clean Ge surface was obtained. The binding energy of the *d*_5/2_ component of Ge(0) peak was observed at 29.0 eV^18^. The Ge 3*d* peak from N_2_^+^ implanted Ge, according to the three components of the different chemical states indicated by Ge-N(I), Ge-N(II), and Ge(0), are shown. The binding energies of the *d*_5/2_ component of Ge-N(I) and Ge-N(II) are 29.4 and 30.1 eV, respectively^18^. The binding energies for the two new fitted peaks are both in the range expected for Ge nitride formation. During the RTA process, Ge_3_N_4_ corresponding to another broad peak at 31.0 eV clearly appears^19^. From these results, we conclude that the significantly increased intensity of the N 1 *s* core-level peak after RTA in Fig. 1(b) is due to the formation of Ge_3_N_4_. The RTA procedure probably causes diffusion of existing molecular N_2_ to form a stable Ge_3_N_4_ layer.

The N 1 *s* core-level spectra in Fig. 1(b) show the effectiveness of Ne^+^ sputtering that completely eradicated the surface nitride of the “as-received” sample. After the N_2_^+^ implantation, an N 1 *s* core-level peak appeared at 397.1 eV, which is a typical binding energy for nitrides^22^. The Ge nitride is formed near the surface. After RTA, the peak position of the N 1 *s* core-level spectrua showed a slight shift towards a higher binding energy with a significantly increased intensity. This indicates that the annealing has caused trapped N_2_ molecules to diffuse out to the surface from within the bulk. The peak is assumed to have come from the mixed chemical states of both Ge-nitride (GeN_x_) and Ge_3_N_4_.

**Figure 2:**
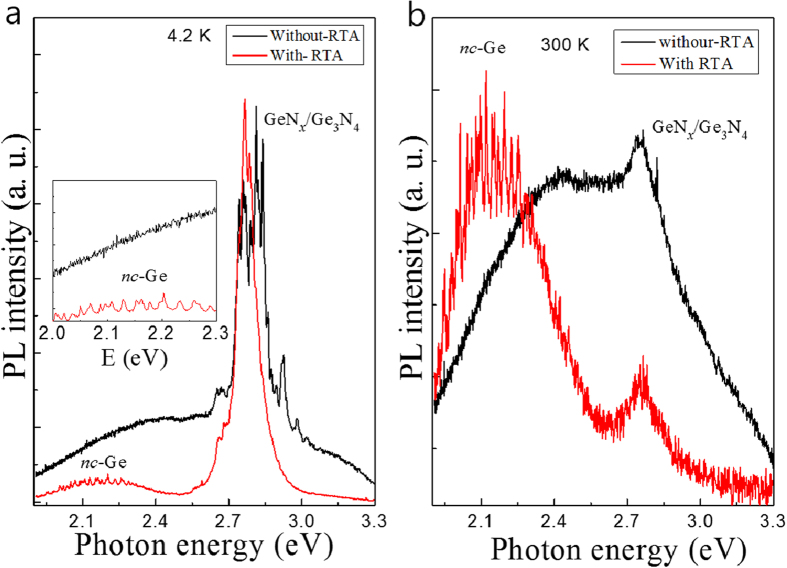
Photoluminescence of the samples with (red) and without (black) RTA measured at 4.2 K (**a**) and 300 K (**b**). Inset depicts the enlarged PL spectra of the low energy region at 4.2 K.

Figure 2(a,b) show the μ-PL spectra of the samples with and without RTA treatment measured at 4.2 and 300 K, respectively. Strong emission lines near 2.8 eV, which are related to Ge_3_N_4_ were observed at 4.2 K for the both samples. The band gap of β-Ge_3_N_4_ allows us to deduce that the direct transition should be around 3.07 eV^23^. The PL observed at around 2.8 eV is energetically far from the expected impurity bound excitonic transitions, which should lie just below band edge of the Ge_3_N_4_. The crystal structure has been confirmed by transmission electronic microscopy to be of poly/amorphous type Ge_3_N_4_ (see supporting infromation Fig. S1). Therefore we suspect that the emission lines originate from defect related emission of the Ge_3_N_4_. Multiple emission lines were observed above the noise level around 2.15 eV at 4.2 K. These lines are thought to be related to *nc*-Ge, for the sample that had undergone RTA treatment (see inset for more detail). Such visible emission from Ge nanocrystals of a few nm in size has previously been found in literature^4^,^11^,^12^,^13^,^14^,^15^. However previously PL spectra from nc-Ge have only ever been reported to show a broad FWHM of about a few hundred meV due to ensemble effects and never resolved as individual spectral lines. The multiple sharp lines in our data become clearer with increasing signal contrast when the temperature is raised up to 300 K. The intensities of the Ge_3_N_4_-related emission lines decrease, allowing the *nc*-Ge related emission lines to become the dominant peaks at 300 K.

**Figure 3:**
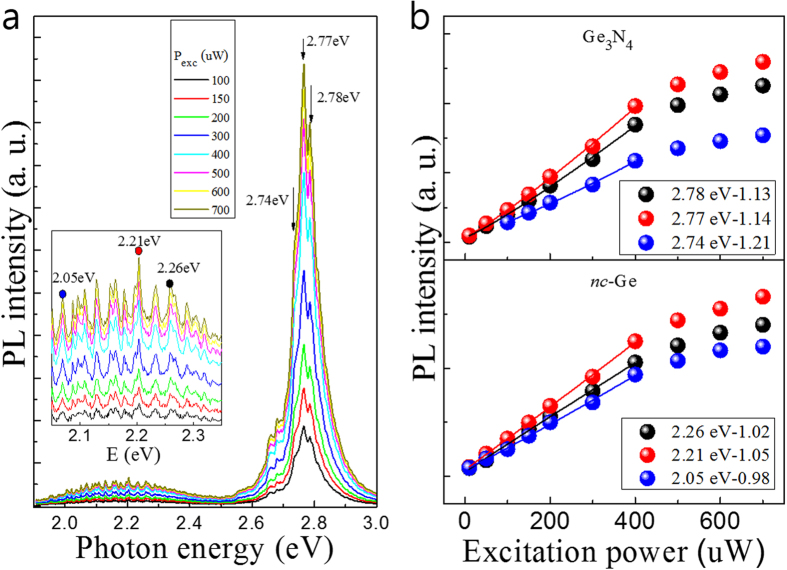
(**a**) Excitation power-dependent photoluminescence spectra of the sample with RTA measured at 4.2 K. Inset depicts the enlarged PL at low energy region. (**b**) PL intensity variation of the representative PL emissions relating to the Ge_3_N_4_ (upper panel) and *nc*-Ge (lower panel) as a function of excitation power, together with their fitted dependencies.

Focusing our study on the RTA sample, Fig. 3(a) shows the excitation power-dependent PL spectra of the sample with RTA treatment. The PL intensities of the *nc*-Ge and Ge_3_N_4_ related emission lines increases approximately linearly with excitation power as expected for excitonic emission. The figure inset shows the zoomed-in spectra around the lower energy region corresponding to the *nc*-Ge. The PL energies relating to *nc*-Ge do not change with increasing excitation power, implying that the emission lines could originate from excitonic transitions. The integrated peak intensities of the Ge_3_N_4_ and *nc*-Ge emissions are presented in the upper and lower pannels of Fig. 2(b) respectively as a function of excitation power. The measurement is fitted with *I* α *P*^*x*^ where *I* is the PL intensity, *P* is the excitation laser power, and *x* is a power parameter defining the slope of the relation, and shown in the legend in Fig. 3(b). The intensity increases linearly and begins to saturate at around 400 μW of laser excitation power. The bottom panel of Fig. 3(b) shows 3 plots for the *nc*-Ge related emssions of excitation power vs. PL intensity. The peaks increases in intensity with excitation power by approximately *I* α *P*^1.0^, as normally found in excitonic emissions, whereas that of the higher energy peak shown in the upper panel of Fig. 3(b) is shown to increase linearly with excitation power and the x value is slightly different from 1, which is not directly related to the excitonic emission but related to deep level related emission of the Ge_3_N_4_. The difference in this proportionality indicates that the emission originates from a different type of transition, and that the 2.7 eV line is not from excitonic emission.

**Figure 4:**
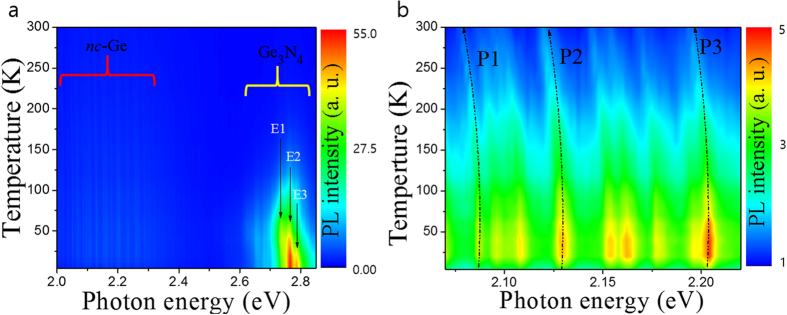
(**a**) Temperature-dependent photoluminescence spectra of the sample with RTA measured at excitation power of 300 μW. (**b**) The mapped PL at low energy region corresponding to *nc*-Ge emission. The dotted lines are added as a guide for the eye.

Figure 4(a) shows the temperature-dependent PL spectra of the sample with RTA measured at a constant excitation power of 300 μW. The PL energy of the Ge_3_N_4_ is independent of temperature indicating that the PL does not follow the usual band gap shrinkage effect. This means that it is possible that the transition is not related to near the band edge emission but rather to a deep level trap, since the energies of the deep levels do not move appreciably as the band edges shift^24^. However, the PL of the *nc*-Ge red-shifts with increasing temperature. Figure 4(b) shows zoomed-in PL spectra mapping of the *nc*-Ge related emissions. A redshift can clearly be observed. In addition, we notice that the PL intesity at lowest temperature of 4.2 K is comparitively weak, but increases in intensity as temperature is raised up to around 40 K.

**Figure 5:**
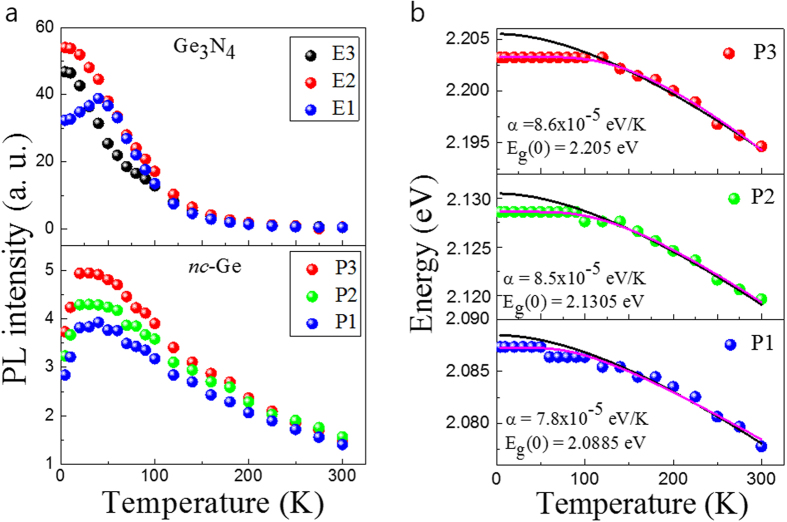
(**a**) PL intensity variation of the representative emission as a function of temperature. The upper and lower panels correspond to the Ge_3_N_4_ and *nc*-Ge, respectively. (**b**) PL energy of the representative emission as a function of temperature. The solid lines were fitted by Varshni’s equation (black solid lines) and O’Donnell and Chen’s equation (pink solid lines).

Results summarising the peak intensity and energy dependence are presented in Fig. 5(a,b), respectively. The PL intensities of the Ge_3_N_4_ peaks decrease with increasing temperature, except for the lowest energy transition, marked E1. This increases in intensity until around 50 K before decreasing at higher temperatures. The PL intensities of the *nc*-Ge related emission lines increases with temperature up to around 40 K and then monotonically decreases, implying that there may exist a trapping level in the Ge_3_N_4_ that can transfer carriers to the excitonic levels of the *nc*-Ge.

Figure 5(b) shows the *nc*-Ge PL peak energy as a function of temperature for three arbitrarily selected peaks labelled P1, P2 and P3. The temperature dependent behaviour is due to band gap shrinkage and is understood in terms of lattice dilation and electron-lattice interactions. This relationship can be described by the semi-empirical Varshni equation^25^: *E*_*g*_(*T*) = *E*_*g*_(0)−α*T*^*2*^/(β + *T*), where *E*_g_(0) corresponds to the energy gap at 0 K, *α* and *β* are known as Varshni’s thermal coefficient and Debye temperature respectively. The experimental data of the *nc*-Ge can be fitted to the above equation (black solid lines) in the high temperature region above 100 K. The Debye temperature was fixed to 374 K based on a reference bulk value^26^. The thermal coefficient *α*, is found to be ~7.2 × 10^−5^ eV/K for the low energy transition peak and slightly increases to 8.9 × 10^−5^ eV/K for the high energy transition peak. This value of *α* is larger by approximately one order of magnitude as compared to that of the bulk semiconductor^27^. It can be seen that the thermal coefficient of the fitted peaks are dependent on their respective emission energy. If we assume that the size of the nanocrystals are directly related to their thermal coefficients, as with silver Ag nanoparticles where a smaller nanoparitcle size and results in a lower thermal coefficient temperature above 50 K^28^. We deduce that the bandgap energy depends on the size of *nc*-Ge, that is, the smaller nanocrystal, the higher transition energy due to confinement effects.

Even though the Varshini fitting matches the data well in the high temperature range, it deviates from the data at low temperatures. A modified relationship for band-gap temperature dependence proposed by O’Donnell and Chen^29^ takes into account the influence of phonons on the bandgap energy to obtain a better fit at for semiconductors at lower temperatures. The following equation is considered: *E*_*g*_(*T*) = *E*_*g*_(0) − *S* 〈*E*_*ph*_〉 [coth(〈*E*_*ph*_〉/2*k*_*B*_*T*) − 1], where 〈*E*_*ph*_〉 is an average phonon energy and *S* is a dimensionless coupling constant. The measured data shows good agreement by fitting with the aforementioned relationship at all measured temperatures (solid pink colors). The fitting parameters of the average phonon energy *E*_*ph*_ were found to be 32.7 meV for P1, 36.4 meV for P2, and 45.1 meV for P3. These values compare reasonably to that of Ge at 37 meV. By considering the energy difference of Ge and the extracted parameters of *nc*-Ge at 0 K, the following values for confinement energy can be obtained; 1.1 meV for P1, 1.9 meV for P2, and 2.29 meV for P3 transitions. The differences in confinement energy suggest the P1, P2 and P3 emissions arise from different sizes of Ge nanocrystal. Another interesting point to note is that the full width at half maximum (FWHM) remains almost unchanged with temperature. It is clear that the PL peaks at 4.2 K have retained their character up to room temperature without a change in shape, and subsequently merge with the PL peaks from neighboring emissions (see supporting information Fig. S3).

**Figure 6:**
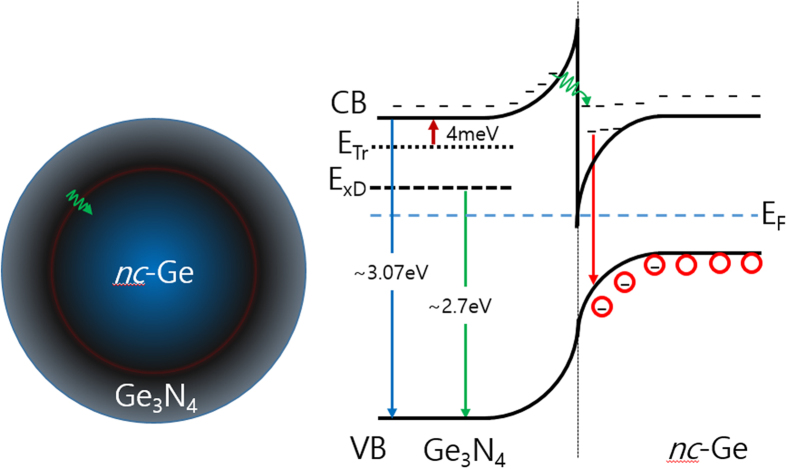
Schematic band diagram of *nc*-Ge surrounded by Ge_3_N_4_. CB and VB represent conduction and valence band, respectively. E_Tr_ and E_xD_ represent the trap and deep level in Ge_3_N_4_, respectively.

Figure 6 shows a proposed schematic band diagram for the *nc*-Ge surrounded by Ge_3_N_4_. For simplicity the *nc*-Ge and Ge_3_N_4_ are drawn as a sphere. Many traps exist in the amorphous Ge_3_N_4_ due to the unsaturated bonds which may lead to large defect concentrations^23^. The deep trap and trapping level of the Ge_3_N_4_ are around 370 meV and 4 meV below conduction band minimum. As temperature increases, the carriers trapped in the trapping level move to the conduction band of the Ge_3_N_4_, and subsequently tunnel to the notch of the *nc*-Ge confining the carriers. Excitons can thus be localized near the surface of the Ge nanocrystals causing fine structures which do not correspond to phonon structures in the Ge. This mechanism can explain the increase of the PL intensity with increasing temperature. If a certain particle is smaller than the exciton Bohr radius, then the excited electron and hole are tightly confined^30^. The effective Bohr radius and binding energy for Wannier excitons in Ge was calculated to be about 22.6~38.8 nm and 1.1~1.8 meV respectively which is remarkably similar to our values obtained from *nc*-Ge^15^.

## Conclusions

In summary, we have investigated the optical emissions of the Ge nanocrystal (*nc*-Ge) through temperature and power-dependent photoluminescence studies. The *nc*-Ge was surrounded by Ge_3_N_4_ prepared by N_2_^+^ ion implantation onto the Ge (111) single crystal. The formation of the *nc*-Ge and Ge_3_N_4_ were confirmed by transmission electron microscopy and X-ray photoemission spectroscopy. Individual PL peaks are finely resolved with our micro-PL system, attributed to emission of the *nc*-Ge. These peaks are shown to be behave with excitonic characteristics and we argue that the spread of *nc*-Ge peaks in the PL spectrum is due to different confinement energies arising from the variation in size of the nanocrystals. Through temperature-dependent studies, we conclude that the emission originating from the *nc*-Ge is from confined carriers with confinement energy of the order of ~1 meV, depending on nanocrystal size.

## Method

### Sample fabrication

p-type Ge (111) single crystal surface (Nilaco Corporation) was initially cleaned by Ne^+^ ion (high purity 99.999%) sputtering for 3 hours with a beam energy of 1 kV under a chamber base pressure of 1.0 × 10^−6^ Torr. The sputtering method was applied until the O 1 *s* and C 1 *s* core-level peaks, at 650 eV, of the incident photon energy measured by HRXPS had disappeared^5^. The N_2_^+^ implantation was done in N_2_ gas (high purity 99.999%) for 1 hour with ion beam energy of 2 kV using the same pressure. After N_2_^+^ implantation, 2 minutes of rapid thermal annealing was carried out at 700 °C in the chamber with a base pressure of 1.0 × 10^−9^ Torr.

### Material characterization

The HRXPS spectra were obtained using synchrotron radiation at 10D beamline of Pohang Accelerator Laboratory. The photon energy was varied from 500 eV (for N 1 *s* core-level) to 80 eV (for Ge 3*d* core-level and valence) to obtain high-quality XPS spectra. The photoelectron signals were recorded with a PHOIBOS 150 electron energy analyzer equipped with a two-dimensional charge-coupled device (2D CCD) detector (Specs GmbH), collecting the photoelectrons normal to the surface. The binding energy scale was calibrated with the Au 4*f* core-level peak at 84.0 eV^18^. The base pressure of the main chamber was maintained below 1.2 × 10^−10^ Torr. Optical properties were intestigated by micro PL with a frequency-tripled femtosecond Ti:sapphire laser (100 fs pulses at 76 MHz) operating at 266 nm as an excitation source, chosen to excite above the bandgap of both the *nc*-Ge and Ge_3_N_4_. A 36× reflecting objective was mounted on a piezo-stage held above the cryostal to both focus the incident laser beam to a spot size of ~0.8 μm^2^, and also to collect the resulting luminescence in the same area in a confocal geometry. The luminescence was then dispersed by a 600 l/ mm reflective grating in a 0.3 m spectrometer and detected using a cooled charge coupled device (CCD) which results in a spectral resolution of ~700 μeV and a spatial resolution of 0.8 μm. The detailed formation of the *nc*-Ge and Ge_3_N_4_, was investigated by HR-TEM at 200 kV acceleration voltage (see supporting information Fig. S1).

## Additional Information

**How to cite this article**: Park, Y. *et al.* Carrier trapping and confinement in Ge nanocrystals surrounded by Ge_3_N_4_. *Sci. Rep.* **6**, 25449; doi: 10.1038/srep25449 (2016).

## Supplementary Material

Supplementary Information
