# Supplementary material for: Carrier trapping and confinement in Ge nanocrystals surrounded by Ge3N4
Source: Sci Rep. 2016 May 5;6:25449. doi: 10.1038/srep25449 (PMC4857120; doi:10.1038/srep25449)
Supplement: Supplementary Information [file srep25449-s1.pdf]

Supporting information for

Carrier trapping and confinement in Ge nanocrystals surrounded by Ge_3N_4

Youngsin Park[1], Christopher C. S. Chan[2], Benjamin P. L. Reid[2], Luke Nuttall[2], Robert A. Taylor[2], Nam-Suk Lee[3], Young Mi Lee[4]

[1]School of Natural Science, Ulsan National Institute of Science and Technology (UNIST), Ulsan 44919, Korea

[2]Clarendon Laboratory, Department of Physics, University of Oxford, Oxford, OX1 3PU, UK

[3]National Institute of Nanomaterials Technology (NINT), Pohang University of Science and Technology, Pohang 37673, Korea

[4]Beamline Division, Pohang Accelerator Laboratory, Pohang 37673, Korea

Figure S1a shows a cross-sectional TEM image of a *nc*-Ge nanocrystal grown on a Ge crystal after rapid thermal annealing (RTA). Nanosized Ge crystals were observed on the Ge surface. For comparison, a TEM image of the sample without RTA is depicted in the inset, where no Ge nanocrystals are evident. Figure S1b shows a zoomed high resolution TEM image of the *nc*-Ge squared region in Fig. 1a. A hemispherical shaped Ge nanostructure is seen on the Ge crystal, and it is surrounded by Ge_3N_4. It is clear that a Ge nitride (G_3eN_4) layer with a thickness of ~4 nm not only covers the *nc*-Ge but also exists between the *nc*-Ge and the Ge substrate, implying that each individual *nc*-Ge is fully isolated structurally. The average heights and diameters of the *nc*-Ge are deduced to be ~18.8 nm and ~25 nm, respectively. Lattice fringes were observed clearly, indicating a high degree of crystallinity for the *nc*-Ge. The inset

in Fig. S1b depicts a selective area diffraction (SAD) pattern of the *nc*-Ge region. The pattern shows one set of 6-fold symmetry, confirming that the nanostructure is indeed *nc*-Ge.

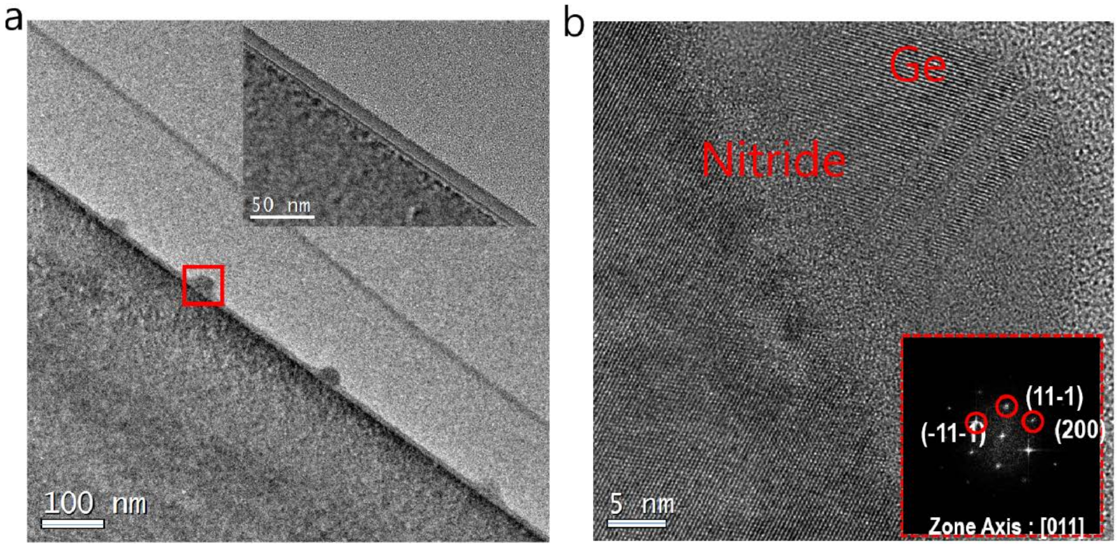

Figure S1. (a) Transmission electron microscopy images of Ge nanocrystals grown on a Ge substrate after RTA. (b) high-resolution image of the red-squared region in (a). Inset depicts a selective area diffraction pattern.

Fig. S2 shows the valence band spectra of the sample at different stages of treatment. The bottom spectrum is obtained from the as-received sample. The Ne^+ sputtering revealed spectra for a clean Ge surface. After N_2^+ implantation, the valence features were found to be significantly broadened. Finally, the RTA simple process facilitated outward diffusion of the nitrogen and chemical reactions such as the formation Ge_3N_4.

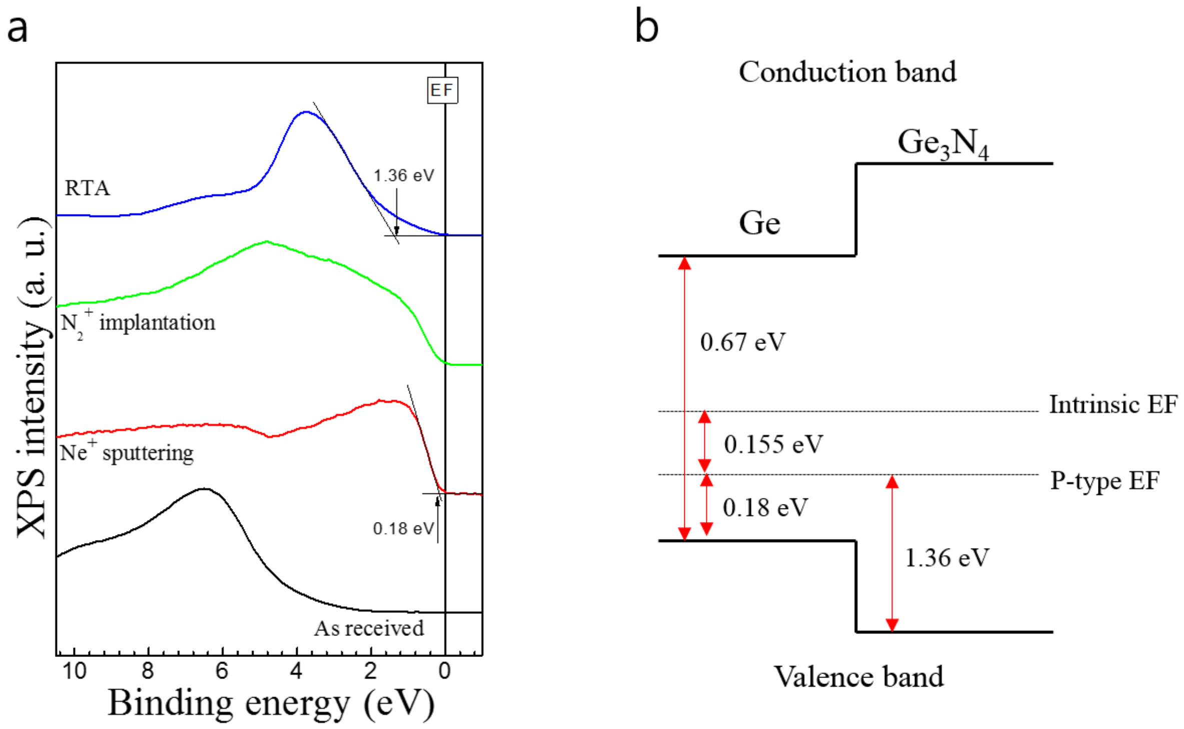

Figure S2. (a) Valence band spectra of pure Ge, Ne^{+} sputtering, N_2^{+} implantation, and rapid thermal annealing samples. (b) Schematic band diagram of the Ge and Ge_3N_4 based on (a)

Fig. S3 shows the PL spectra at representatively selected temperatures. In particular, the P2 peak was fitted by a Gaussian function. The full width at half maximum (FWHM) at each temperature does not change, indicating that phonon effects are less important as the temperature increases. The average FWHM is about 7.4 meV.

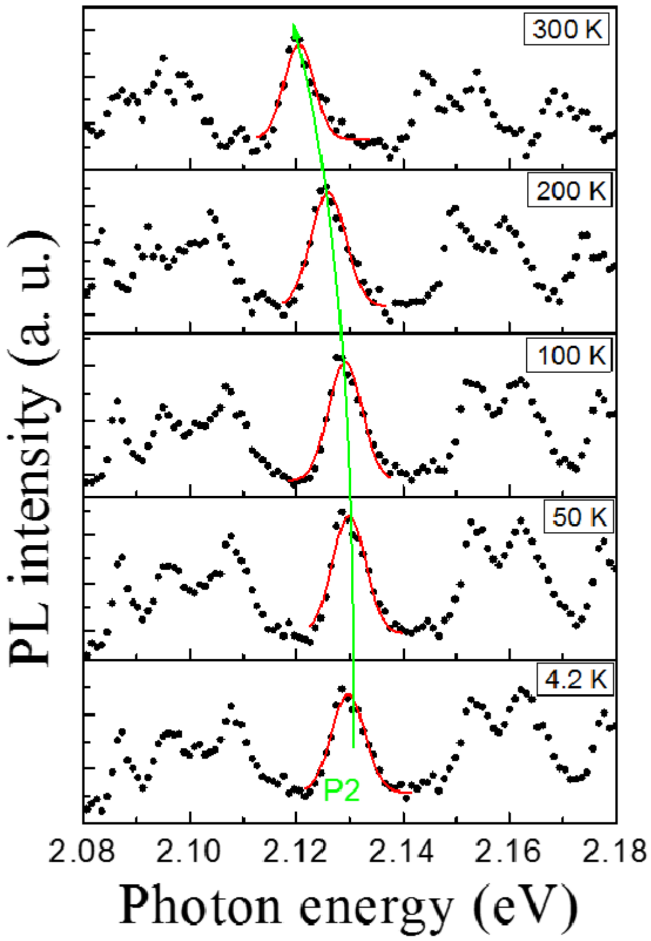

Figure S3. Temperature dependent PL spectra at selected temperatures of 4.2, 50, 100, 200, and 300 K. The black circles are measured data and the red curve is a fit using a Gaussian function. The green line is a guide to the eye to demonstrate the peak energy shift as a function of temperature.
